# Discovery of two novel and adjacent QTLs on chromosome B02 controlling resistance against bacterial wilt in peanut variety Zhonghua 6

**DOI:** 10.1007/s00122-020-03537-9

**Published:** 2020-01-24

**Authors:** Huaiyong Luo, Manish K. Pandey, Ye Zhi, Huan Zhang, Siliang Xu, Jianbin Guo, Bei Wu, Haiwen Chen, Xiaoping Ren, Xiaojing Zhou, Yuning Chen, Weigang Chen, Li Huang, Nian Liu, Hari K. Sudini, Rajeev K. Varshney, Yong Lei, Boshou Liao, Huifang Jiang

**Affiliations:** 1 Key Laboratory of Biology and Genetic Improvement of Oil Crops, Ministry of Agriculture, Oil Crops Research Institute of the Chinese Academy of Agricultural Sciences (CAAS), Wuhan, 430062 China; 2 International Crops Research Institute for the Semi-Arid Tropics (ICRISAT), Hyderabad, 502324 India; 3 Angel Yeast Co., Ltd, Yichang, 443003 Hubei China

## Abstract

**Key message:**

Two novel and adjacent genomics and candidate genes for bacterial wilt resistance were identified on chromosome B02 in peanut variety Zhonghua 6 using both traditional QTL mapping and QTL-seq methods.

**Abstract:**

Peanut (*Arachis hypogaea*) is an important oilseed crop worldwide. Utilization of genetic resistance is the most economic and effective approach to control bacterial wilt, one of the most devastating plant diseases, in peanut production. To accelerate the genetic improvement of bacterial wilt resistance (BWR) in peanut breeding programs, quantitative trait locus (QTL) mapping has been conducted for two resistant varieties. In this context, we deployed linkage mapping as well as sequencing-based mapping approach, QTL-seq, to identify genomic regions and candidate genes for BWR in another highly resistant variety Zhonghua 6. The recombination inbred line population (268 progenies) from the cross Xuhua 13 × Zhonghua 6 was used in BWR evaluation across five environments. QTL mapping using both SSR- and SNP-based genetic maps identified a stable QTL (*qBWRB02-1*) on chromosome B02 with 37.79–78.86% phenotypic variation explained (PVE) across five environments. The QTL-seq facilitated further dissection of *qBWRB02-1* into two adjacent genomic regions, *qBWRB02-1-1* (2.81–4.24 Mb) and *qBWRB02-1-2* (6.54–8.75 Mb). Mapping of newly developed Kompetitive allele-specific PCR (KASP) markers on the genetic map confirmed their stable expressions across five environments. The effects of *qBWRB02-1-1* (49.43–68.86% PVE) were much higher than *qBWRB02-1-2* (3.96–6.48% PVE) and other previously reported QTLs. Nineteen putative candidate genes affected by 49 non-synonymous SNPs were identified for *qBWRB02-1-1*, and ten of them were predicted to code for disease resistance proteins. The major and stable QTL *qBWRB02-1-1* and validated KASP markers could be deployed in genomics-assisted breeding (GAB) to develop improved peanut varieties with enhanced BWR.

**Electronic supplementary material:**

The online version of this article (10.1007/s00122-020-03537-9) contains supplementary material, which is available to authorized users.

## Introduction

The cultivated peanut (*Arachis hypogaea* L.), also known as groundnut, is an important legume crop grown in more than 100 countries with 47.10 million tonnes global production in 2017 (FAOSTAT 2019). It is consumed worldwide in the form of oil, nuts, peanut butter, candy, etc. (Varshney et al. 2013). Bacterial wilt disease poses a serious threat to peanut production in China, the highest peanut producer in the world, in addition to Vietnam, Indonesia, Uganda and other Southeast Asian countries. This disease usually causes 10–30% yield losses and can cause up to 50–100% in severe circumstances (Jiang et al. 2017). It is caused by the soil-borne bacterium *Ralstonia solanacearum* which has a wide host range including over 450 plant species belonging to more than 50 botanical families (Deslandes and Genin 2014; Salanoubat et al. 2002).

Deploying host resistance is the most economical and eco-friendly approach to control bacterial wilt disease in peanut production. To do so, it is very important to identify stable resistance sources with high survival rates under heavy bacterial wilt disease pressure based on multi-environment disease screening (Mallikarjuna and Varshney 2014). More than 140 peanut landraces, improved varieties or wild *Arachis* species were identified to possess high levels of resistance to bacterial wilt (Liao 2017). Cultivation of resistant varieties has greatly reduced yield loss in peanut production to less than 5%. However, the breeding of new resistant varieties faces challenges due to the inability of conducting large-scale screening in conventional breeding programs (Jiang et al. 2017). Under such circumstances, it is essential to identify QTLs and linked markers for bacterial wilt resistance (BWR), which could be deployed in genomics-assisted breeding (GAB) to accelerate the breeding process (Janila et al. 2016; Varshney et al. 2014).

Up to now, QTL mapping has been conducted for only two resistant varieties, i.e., Yueyou 92 and Yuanza 9102, through either traditional genetic map-based QTL mapping or sequencing-based mapping approaches. Based on a 1627.4 cM genetic map containing 237 SSR and SNP markers, Zhao et al. (2016) identified a major QTL *qBW-1* for BWR in an F_2_ population and its recombination inbred line (RIL) population derived from the crossing of Yueyou 92 and susceptible variety Xinhuixiaoli. The resistance in Yueyou 92 is from a Chinese landrace Xiekangqing, which is a major resistance source utilized for bacterial wilt disease in Chinese breeding programs (Mallikarjuna and Varshney 2014). In addition to traditional genetic map-based QTL mapping, sequencing-based mapping approaches, such as QTL-seq (Takagi et al. 2013), facilitate high resolution and faster mapping of genomic regions and candidate genes for target traits in crop plants (Pandey et al. 2016; Wambugu et al. 2018; Zhong et al. 2018). Based on the genome sequences of *A. duranensis* and *A. ipaensis* (the diploid ancestors of allotetraploid cultivated peanut) (Bertioli et al. 2016), the QTL-seq approach was deployed for the first time in a RIL population and helped in rapid discovery of the major and stable QTL *qBWRB02.1* for BWR in Yuanza 9102 as well as its candidate genes and diagnostic markers (Luo et al. 2019). The resistance source for Yuanza 9102 was suggested to be a resistant wild species *A. diogoi* (Mallikarjuna and Varshney 2014). More importantly, the resistant allele of *qBWRB02.1* was absent from other resistant peanut genotypes according to diagnostic markers genotyping (Luo et al. 2019). Therefore, more efforts are needed to discover the QTLs for BWR in other resistant genotypes using either traditional genetic map-based QTL mapping or sequencing-based mapping approaches, which would increase the diversity of utilizable resistant QTLs/genes in peanut breeding and might improve the resistance level by integrating multiple resistance alleles.

The improved peanut variety Zhonghua 6 has been highly and stably resistant to bacterial wilt disease since released by the Oil Crops Research Institute of the Chinese Academy of Agricultural Sciences in 2000. Its resistance derives from the Chinese landrace Taishan Zhenzhu, which is highly resistant but seldom used in peanut breeding because of undesirable features such as small-seeded and low-yield. Compared to Taishan Zhenzhu, Zhonghua 6 has improved agronomic traits thus would be an important resistance source in future breeding programs. In the present study, a recombination inbred line (RIL) population (268 progenies) developed from the cross of Xuhua 13 (highly susceptible) × Zhonghua 6 was used to discover the genetic basis of BWR in Zhonghua 6. More SSR loci were added to the previously published SSR-based genetic map (Luo et al. 2018) in the present study, and QTL mapping were conducted with phenotyping data across five environments and SSR- and SNP-based genetic map. In addition, based on the recently published genome sequence of cultivated peanut (Chen et al. 2019), the QTL-seq approach was deployed as well to identify the genomic regions, candidate genes and efficient markers for BWR in the present study.

## Materials and methods

### Plant materials

The improved peanut variety Xuhua 13 is highly susceptible to bacterial wilt disease, while Zhonghua 6 has been highly and stably resistant since it was released. A RIL population (268 lines) was developed from the cross Xuhua 13 × Zhonghua 6 following single seed decent (SSD) method (Luo et al. 2018). Our previous study reported SSR-based genetic mapping using a subset (187 RILs) of the mapping population leading to development of a genetic map with 817 SSR loci (Luo et al. 2018). In the present study, DNA was extracted from the same subset in the F_5_ generation for genotyping of SSR markers to improve the genetic map. Recently, 186 of the 187 RILs in the F_6_ generation were genotyped by ddRADseq (double-digest Restriction-site Associated DNA sequencing), upon which a SNP-based genetic map was constructed with 2595 SNPs, spanning 2465.62 cM with an average inter-marker distance of 0.95 cM (Liu et al. 2019). Three generations (F_6_–F_8_) of the RIL population (268 lines) were used for phenotyping BWR. The F_9_ generation of the RIL population (268 lines) was used to extract DNA for the genotyping of newly developed Kompetitive Allele-Specific PCR (KASP) markers.

### Phenotyping and statistical analysis of bacterial wilt resistance

The BWR of peanut materials were assessed in two disease nurseries under heavy pressure in Hongan (31.36° N, 114.61° E, constructed in 1964) and Nanchong, China (30.67° N, 106.06° E, constructed in 2007). These disease nurseries, in which bacterial wilt disease naturally and evenly occurs across years, were utilized in the BWR evaluation of our previous study (Luo et al. 2019). The RIL population (Xuhua 13 × Zhonghua 6) was planted in a randomized block design with three replications in Hongan during 2015 (F_6_ generation), 2016 (F_7_ generation) and 2017 (F_8_ generation) as well as in Nanchong during 2015 (F_6_ generation) and 2017 (F_8_ generation). These five environments were designated as HA2015, HA2016, HA2017, NC2015 and NC2017, respectively. The percentage of plants that were not killed by bacterial wilt disease during the entire period was used to indicate the resistance and referred as survival rate (Luo et al. 2019). Analysis of variance (ANOVA) was performed with the IBM SPSS Statistics version 22 software. The broad-sense heritability for BWR was estimated based on plot mean and entry mean, respectively (Luo et al. 2017).

### The improvement of the SSR-based the genetic map

To improve the previously published SSR-based genetic map (Luo et al. 2018), 456 SSR markers that exhibited polymorphism between Xuhua 13 and Zhonghua 6 were used to genotype the same 187 RILs in F_5_ generations. These markers were selected according to their positions in the reference genomes of *Arachis* (Bertioli et al. 2016, 2019) to increase density of the previously published SSR-based genetic map (Luo et al. 2018). PCR amplification and visualization were conducted with the same approach of the previous study (Luo et al. 2018). Similarly, genetic map was re-constructed using the JoinMap 4.0 (Van Ooijen 2006) and visualized with the MapChart 2.3 software (Voorrips 2002). The Kosambi mapping function was used to estimate genetic distances (Kosambi 1943).

### QTL mapping using SSR- and SNP-based genetic maps

Using the improved SSR-based genetic map and the recently reported SNP-based genetic map (Liu et al. 2019), genome-wide QTL mapping for BWR was performed using the mean value of survival rates in three replicates in each environment. QTL analysis was conducted by the composite interval mapping (CIM) method using the Windows QTL Cartographer 2.5 software (Wang et al. 2012). The threshold of LOD for declaring the presence of a QTL was determined by 1000 permutation tests. QTLs are designated with an initial letter “q” followed by the trait name (BWR), and the corresponding chromosome, similar to the previously described nomenclature (Luo et al. 2019). The codes “− 1” and “− 2” were added for QTLs detected on the same chromosome. QTLs with PVE higher than 10% were considered as major otherwise as minor. In addition, a QTL was considered as stable if it was identified across five environments.

### Identification of genomic regions for bacterial wilt resistance with the QTL-seq approach

The QTL-seq approach (Takagi et al. 2013) was adopted to identify the genomic regions for BWR following the pipeline of the previous study (Luo et al. 2019) with a few modifications (as shown in Figure S1). Briefly, 25 RILs with extremely high or low mean survival rates were used to construct the resistant bulk (RB) or susceptible bulk (SB), respectively. Pair-ended reads (150 bp) were generated using the Illumina Hiseq platforms for RB, SB, susceptible parent (SP) and resistant parent (RP). The genome assembly of the cultivated peanut Fuhuasheng (Chen et al. 2019) was downloaded from the NCBI website and used as the reference to develop reference-guided parental assemblies, i.e., the reference-guided assembly for the susceptible parent Xuhua 13 (the SP assembly) and for the resistant parent Zhonghua 6 (the RP assembly). This was the major difference from the previous study (Luo et al. 2019)*.* The high-quality reads of SB and RB were equalized and aligned to the parental assembly to identify SNPs. The ΔSNP-index for each SNP was calculated by subtracting SNP-index of SB from SNP-index of RB (${\text{SNP-index}} = \frac{{{\text{Count }}\,{\text{of}}\,{\text{alternate}}\,{\text{allele}}}}{{{\text{Total }}\,{\text{read}}\,{\text{count}}}}$). The sliding window analyses for SNP-index and ΔSNP-index were conducted with 1 Mb interval and 50 kb increment to identify genomic regions for BWR.

### Marker development and validation

To validate the identified genomic regions for BWR, KASP markers (Semagn et al. 2014) were developed for the significant SNPs using the genomic sequences of Fuhuasheng (Chen et al. 2019). Each KASP marker was firstly validated with Xuhua 13 and Zhonghua 6 and then used to genotype the 268 lines of the RIL population. The genotyping of KASP markers was conducted at the China Golden Marker (Beijing) Biotech Co., Ltd. In addition, SSR markers within the candidate genomic regions were also used to genotype the 268 RILs. Local genetic maps were constructed with the JoinMap 4.0 software (Van Ooijen 2006) and used to identify QTLs for BWR by both single-marker analysis (SMA) and composite interval mapping (CIM) methods of the Windows QTL Cartographer 2.5 software (Wang et al. 2012).

### Candidate genes analysis

To identify candidate genes, effective SNPs associated with BWR were identified following the same procedure of our previous study (Luo et al. 2019). The SnpEff v3.0 software (Cingolani et al. 2012) was then used to predict the functions of these effective SNPs based on the published annotation of the genomic sequences of Fuhuasheng (Chen et al. 2019). The putative genes affected by non-synonymous SNPs were selected as candidates for the BWR. The function of candidate genes were predicted using the Blast2GO (Gotz et al. 2008) and eggNOG-mapper tool (Huerta-Cepas et al. 2016). The InterPro online server (Finn et al. 2017) was used to predict the compositions of conserved domains encoded by candidate genes.

## Results

### Phenotypic variations of BWR in the RIL population

**Fig. 1:**
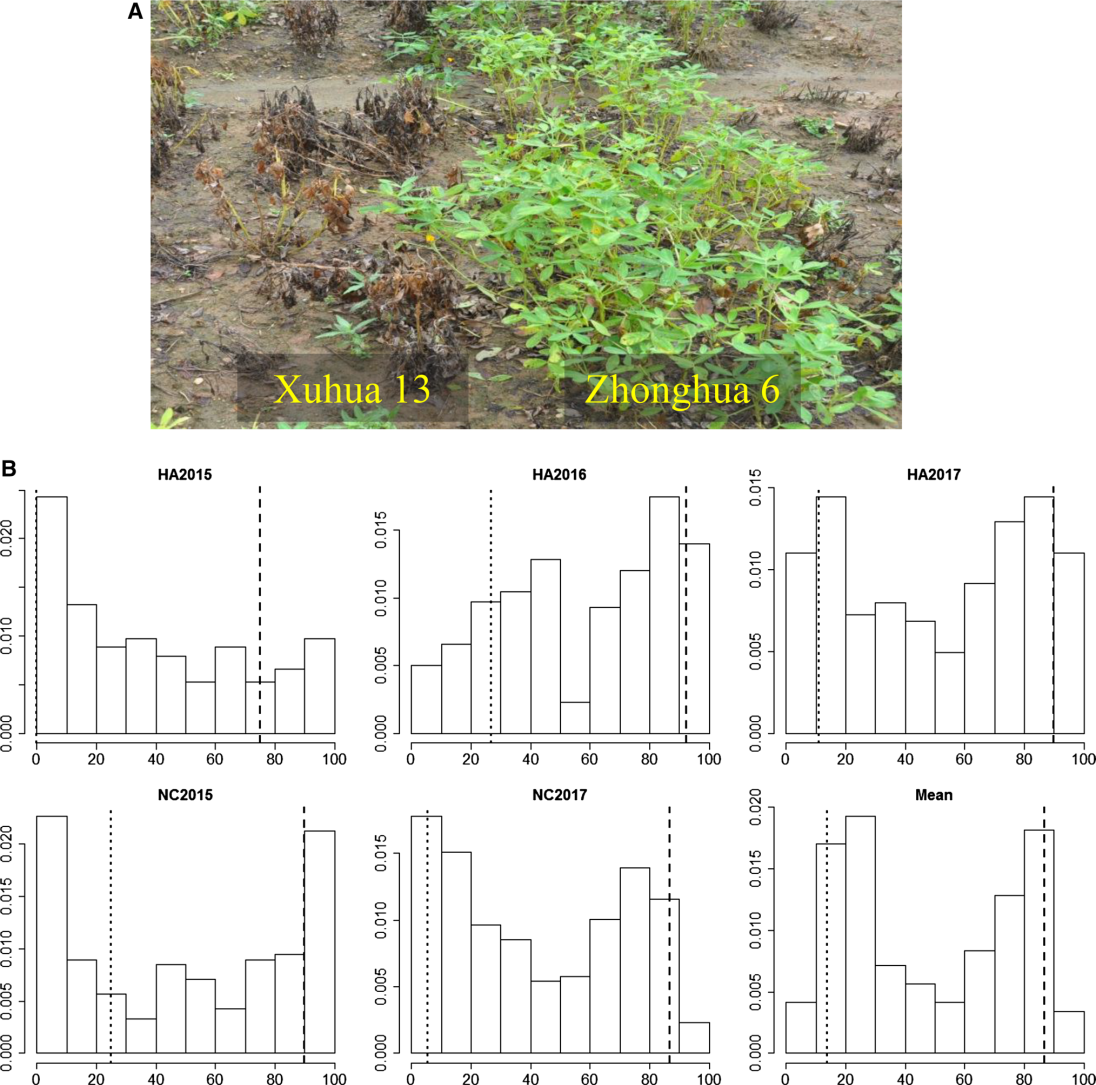
Phenotypic variations of the RIL population for bacterial wilt resistance across five environments. **a** The difference of survival rate of susceptible parent Xuhua 13 and resistant parent Zhonghua 6 in the disease nursery, **b** frequency distribution for survival rates in RIL population. The *y*-axis represented density, while the *x*-axis represented values of survival rates. The dotted line represented the survival rate of Xuhua 13, and the dashed line represented the survival rate of Zhonghua 6. The HA2015, HA2016, HA2017, NC2015 and NC2017 indicated the phenotyping were conducted in Hongan city (HA) during 2015, 2016, 2017 and Nanchong city (NC) during 2015, 2017

The resistant parent Zhonghua 6 (RP) showed significantly higher survival rates (86.75% on average) than the susceptible parent Xuhua 13 (SP) (13.69% on average) across five environments (Fig. 1). The survival rates of the 268 RILs showed continuous distributions with two peaks (Fig. 1b), indicating the existing of major QTLs for BWR. Analysis of variance revealed that genotype, environment and genotype × environment interactions significantly (*P* < 0.001) influenced BWR (Table S1). Broad-sense heritability for BWR was estimated to be 71.07% based on plot mean and 93.72% based on entry mean, indicating strong control by genetic factors.

### Development of high-density SSR-based genetic map

A total of 180 SSR markers (Table S2) were successfully added to the previously reported SSR-based genetic map (Luo et al. 2018). Five markers amplified two genetic loci, while the remaining 175 markers amplified single locus (Table S3). The improved SSR-based genetic map consisted of 1002 loci spanning 1838.10 cM with an average inter-marker distance of 1.83 cM (Figure S2, Table S4). The newly genotyped 185 loci were mapped across genome except chromosomes A01, B01, B03 and B09. The improved SSR-based genetic map had 516 loci for the A subgenome and 486 loci for the B subgenome with map lengths of 948.55 and 889.55 cM, respectively. The length of linkage groups varied from 32.18 cM (A04) to 134.71 cM (B03), and the number of loci ranged from 7 to 105 markers (Table S5).

### Discovery of genome-wide QTLs for BWR using SSR- or SNP-based genetic map

**Fig. 2:**
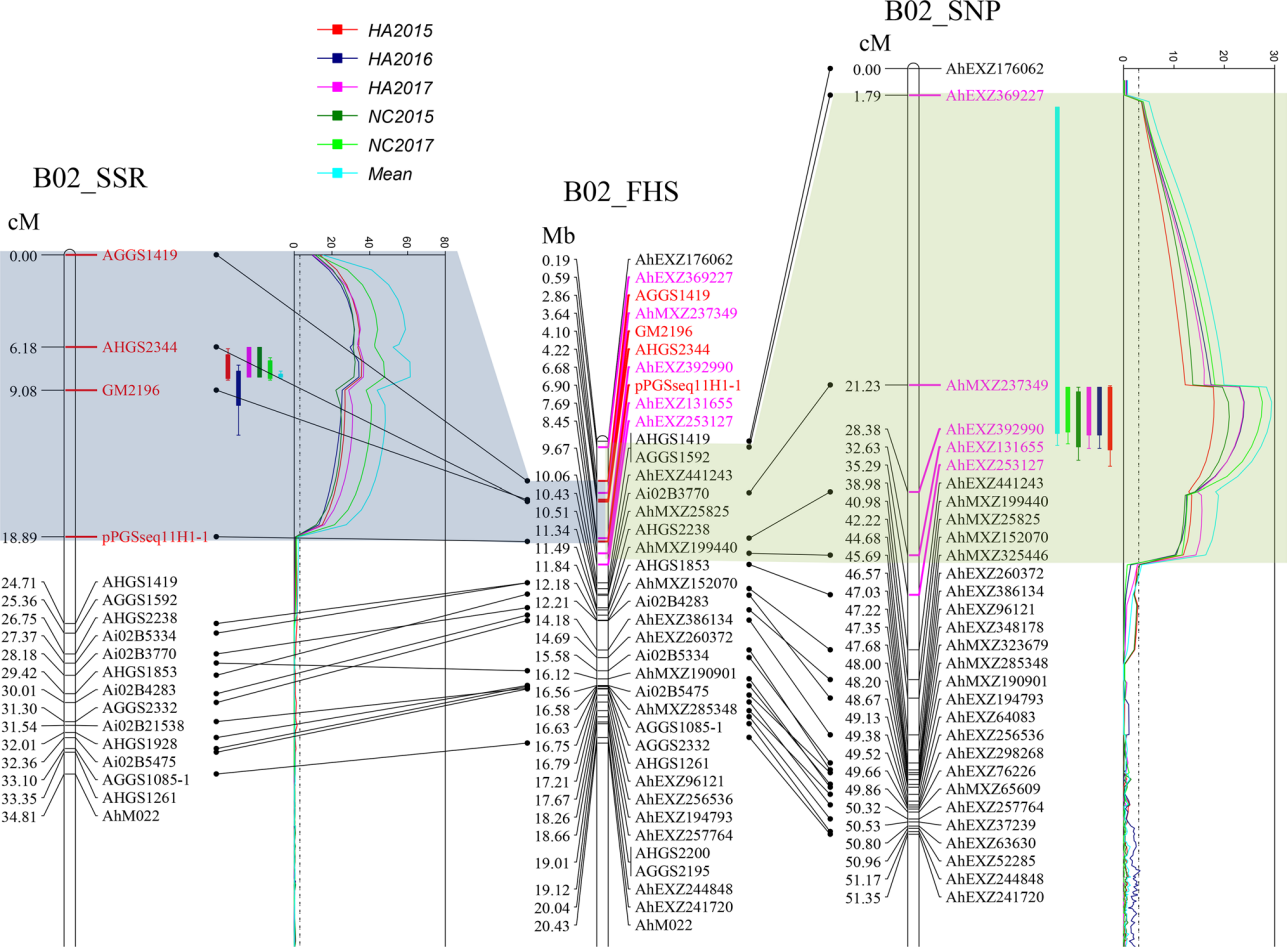
Co-localization of the major QTL identified by SSR- and SNP-based genetic map for bacterial wilt resistance on chromosome B02. B02_SSR indicates the linkage map B02 constructed based on SSR markers. B02_SNP indicates the reversed linkage map B02 constructed based on SNP loci by Liu et al. (2019). B02_FHS indicates the estimated physical positions of SSR and SNP loci on the genomic sequence of chromosome B02 of Fuhuasheng. The positions of QTL intervals were highlighted in blue and green color, respectively (color figure online)

Genome-wide QTL analysis was performed using the improved SSR-based genetic map and phenotypic data of BWR obtained for the subset (187 RILs) of the mapping population in five environments. Through composite interval mapping (CIM), one major QTL was identified on chromosomes B02 while two minor QTLs were identified on B02 and A07 (Table 1, Figure S3). The major QTL, *qBWRB02-1* on the chromosome B02, was stable in expression across five environments with 53.93–78.86% phenotypic variation explained (PVE) while the two minor QTLs, *qBWRB02-2* and *qBWRA07*, were only expressed in the HA2017 environment (3.78–4.31% PVE). According to e-PCR locations of the four flanking SSR markers (AGGS1419, GM2196, AHGS2344 and pPGSseq11H1-1) on the genome assembly of Fuhuasheng (Chen et al. 2019), *qBWRB02-1* was probably located in a 6.90 Mb interval (0–6.90 Mb) on chromosome B02 (Fig. 2).

**Table 1:** QTL mapping for bacterial wilt resistance using the improved SSR-based genetic map

QTL	Env^a^	Chr^b^	Genetic position (cM)	LOD value	Marker interval	Physical interval (Mb)	Additive effect	PVE^c^ (%)
*qBWRB02-1*	HA2015	B02	7.21	35.81	AGGS1419–pPGSseq11H1-1	0–6.90	25.20	60.17
	HA2016	B02	8.21	34.26	AGGS1419–pPGSseq11H1-1	0–6.90	20.46	53.93
	HA2017	B02	7.21	36.84	AGGS1419–pPGSseq11H1-1	0–6.90	23.65	54.56
	NC2015	B02	7.21	32.28	AGGS1419–pPGSseq11H1-1	0–6.90	29.76	58.17
	NC2017	B02	8.21	47.91	AGGS1419–pPGSseq11H1-1	0–6.90	24.49	69.33
	Mean	B02	8.21	61.44	AGGS1419–pPGSseq11H1-1	0–6.90	25.38	78.86
*qBWRB02-2*	HA2017	B02	64.41	4.02	AGGS1635-2–AHGS1241	107.50–111.70	6.22	3.78
*qBWRA07*	HA2017	A07	95.01	4.41	AGGS0259–AGGS2505	161.16–165.52	6.57	4.31

^a^Environment

^b^Chromosome

^c^Phenotypic variation explained

Genome-wide QTL analysis was performed using the recently constructed SNP-based genetic map (Liu et al. 2019) as well. This result confirmed the presence of the major and stable QTL *qBWRB02-1* on chromosome B02 (37.79–53.85% PVE), which was estimated to be located in a 7.51 Mb interval (0.59–8.45 Mb) through BLASTn the RAD tags of five flanking SNPs (AhEX369227, AhMXZ237349, AhEXZ392990, AhEXZ131655 and AhEXZ253127) to the genome sequence of Fuhuasheng (Table 2; Fig. 2). Two additional minor QTLs (3.75% or 5.88% PVE) were identified on chromosome B01 (Table 2; Figure S4). The minor QTL *qBWRB01-1* was identified in the HA2015 environment, while the other minor QTL *qBWRB01-2* was identified using the mean values of survival rates across five environments.

**Table 2:** QTL mapping for bacterial wilt resistance using the SNP-based genetic map

QTL	Env^a^	Chr^b^	Genetic position (cM)	LOD value	Marker interval	Physical interval (Mb)	Additive effect	PVE^c^ (%)
*qBWRB02-1*	HA2015	B02	91.11	17.98	AhEXZ253127–AhEXZ369227	0.59–8.45	19.80	37.79
	HA2016	B02	91.11	23.94	AhEXZ253127–AhEXZ369227	0.59–8.45	19.44	45.14
	HA2017	B02	91.11	23.84	AhEXZ253127–AhEXZ369227	0.59–8.45	21.28	45.73
	NC2015	B02	91.11	20.98	AhEXZ253127–AhEXZ369227	0.59–8.45	26.68	47.80
	NC2017	B02	91.11	27.57	AhEXZ253127–AhEXZ369227	0.59–8.45	21.36	51.88
	Mean	B02	91.11	29.39	AhEXZ253127–AhEXZ369227	0.59–8.45	21.08	53.85
*qBWRB01-1*	HA2015	B01	48.51	4.81	AhEXZ281542–AhMXZ205274	83.92–84.88	10.30	5.88
*qBWRB01-2*	Mean	B01	45.41	4.48	AhEXZ117519–AhMXZ288911	62.04 –65.73	7.19	3.75

^a^Environment

^b^Chromosome

^c^Phenotypic variation explained

Therefore, based on the comparison between intervals identified by SSR- and SNP-based genetic maps, the major and stable QTL *qBWRB02-1* was estimated to be located within a 6.31 Mb physical distance (0.59–6.90 Mb, between AhEXZ369227 and AhEXZ392990) on chromosome B02 (Fig. 2).

### Identification of genomic regions for BWR by QTL-seq using the tetraploid reference genome

**Fig. 3:**
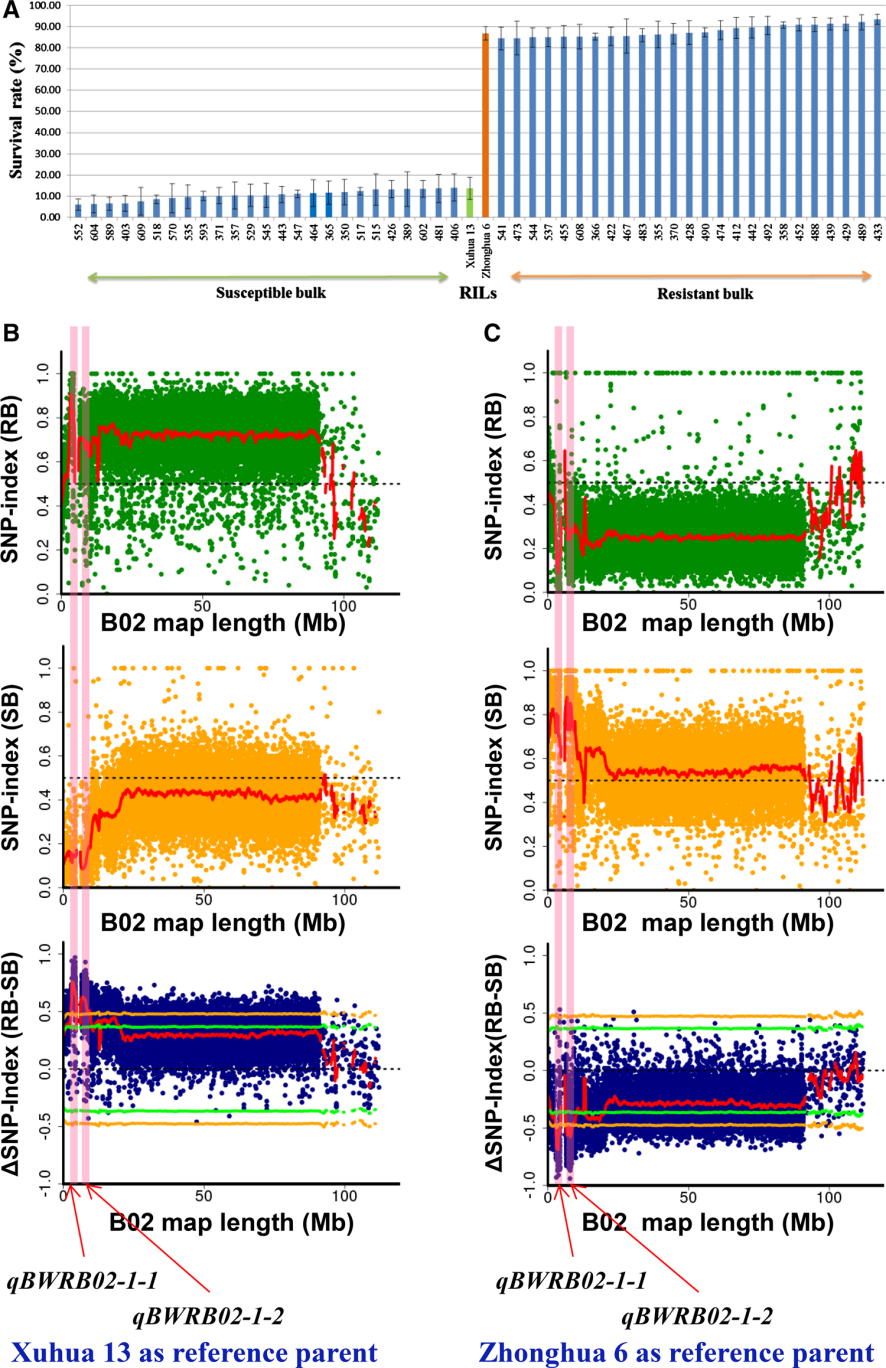
Construction of extreme bulks and identification of genomic regions for bacterial wilt resistance by the QTL-seq approach. **a** Phenotypic variability among the RILs selected for development of extreme bulks for bacterial wilt resistance. Based on mean values of the five environments, 25 RILs with lowest survival rates and 25 RILs with highest survival rates were used to constitute susceptible and resistant bulks, **b** genomic regions for bacterial wilt resistance identified by QTL-seq using the Xuhua 13 as reference, **c** genomic regions for bacterial wilt resistance identified by QTL-seq using the Zhonghua 6 as reference

Based on the mean survival rates of the whole population (268 RILs) across five environments, 25 RILs with survival rates of 5.99–14.37% and 25 RILs with survival rates of 84.93–95.42% were used to prepare the susceptible bulk (SB) and resistant bulk (RB), respectively (Fig. 3a). Whole-genome resequencing data, including 936.70 million reads (140.51 Gb) for susceptible parent (SP, Xuhua 13), 527.10 million reads (79.06 Gb) for resistant parent (RP, Zhonghua 6), 967.16 million reads (145.07 Gb) for RB and 962.28 million reads (144.34 Gb) for SB (Table 3; Table S6), were generated using Illumina platforms.

**Table 3:** Summary of survival rates and Illumina sequencing of parental lines and bulks

Sample	Mean survival rates (%)	Number of reads generated	Total bases	Genome coverage (%)	Mean depth (*X*)
Xuhua 13^a^	13.69	936,700,922	140,505,138,300	96.75	26.79
RB^b^	87.86	967,161,084	145,074,162,600	97.20	38.21
SB^b^	10.47	962,279,282	144,341,892,300	97.19	36.74
Zhonghua 6^a^	86.75	527,095,770	79,064,365,500	96.35	18.89
RB^c^	87.86	967,161,084	145,074,162,600	97.20	38.19
SB^c^	10.47	962,279,282	144,341,892,300	97.19	37.12

^a^The short reads of parental lines were aligned to the published genome sequences of Fuhuasheng

^b^The short reads of the extreme bulks were aligned to the SP “reference sequence” developed by replacement of SNPs between Xuhua 13 and Fuhuasheng

^c^The short reads of bulks were aligned to the RP “reference sequence” developed by replacement of SNPs between Zhonghua 6 and Fuhuasheng

Initially, a reference-guided assembly was developed for Xuhua 13 by aligning its reads (96.75% genome coverage and 26.79 mean read depth) to the genome assembly of Fuhuasheng (Chen et al. 2019), which was then referred to as the SP assembly (Figure S1). The reads of the RB and SB were mapped to the SP assembly, and achieved 97.20% mapping coverage and 38.21 mean read depth for the RB while 97.19% coverage and 36.74 mean read depth for the SB (Table 3, Table S6). A total of 303,290 genome-wide SNPs were identified (Table S7). Based on the sliding window analysis of their SNP-indexes and ΔSNP-indexes, two major peaks on chromosome B02 were identified for BWR at a statistical confidence of *P* < 0.01, spanning 1.47 Mb (2.80–4.27 Mb) and 3.54 Mb (6.11–9.65 Mb) intervals, respectively (Fig. 3b, Figure S5–S7). Their ΔSNP-indexes were positive, indicating that more alleles were from the non-reference parent Zhonghua 6 in the RB (Table 4).

**Table 4:** Genomic regions identified for bacterial wilt resistance on chromosome B02 using QTL-seq

Reference assembly	Genomic region (Mb)	Length (Mb)	ΔSNP-index^d^	U99^e^	L99^f^	Allele source
SP^a^	2.80–4.27	1.47	0.76	0.49	− 0.49	Zhonghua 6
	6.11–9.65	3.54	0.63	0.48	− 0.48	Zhonghua 6
RP^b^	2.81–4.24	1.43	− 0.69	0.48	− 0.48	Zhonghua 6
	6.54–8.75	2.21	− 0.58	0.47	− 0.48	Zhonghua 6
Combined^c^	2.81–4.24	1.43				Zhonghua 6
	6.54–8.75	2.21				Zhonghua 6

^a^The susceptible parent Xuhua 13

^b^The resistant parent Zhonghua 6

^c^The genomic regions determined based on the results of SP and RP

^d^The highest **Δ**SNP-index (SNP-index of resistant bulk − SNP index of susceptible bulk) of widows in the identified genomic region

^e^99% confidence interval upper side

^f^99% confidence interval lower side

Similarly, a reference-guided assembly was developed for Zhonghua 6 by aligning its reads (96.35% genome coverage and 18.89 mean read depth) to the genome assembly of Fuhuasheng, which was then referred to as the RP assembly (Figure S1). The reads of the RB and SB were also mapped to the RP assembly and achieved 97.20% coverage and 38.19 mean read depth for the RB while 97.19% coverage and 37.12 mean read depth for the SB (Table 3; Table S6). A total of 320,522 genome-wide SNPs (Table S7) were identified, among which 281,637 SNPs were also identified in the above analysis using the SP assembly as reference. Based on the sliding window analysis, two similar genomic regions on chromosome B02 were identified for BWR, i.e., a 1.43 Mb (2.81–4.24 Mb) interval and a 2.21 Mb (6.54–8.75 Mb) interval (Fig. 3c; Figure S8–S10). The ΔSNP-indexes of the two genomic regions were negative, indicating that more alleles were from reference parent Zhonghua 6 in the RB (Table 4).

When combined the results of both linkage mapping and QTL-seq, the above genomic regions were overlapped with the major and stable QTL *qBWRB02-1*. Therefore, two adjacent genomic regions were selected as the candidates for BWR, i.e., 2.81–4.24 Mb and 6.54–8.75 Mb on B02 for further detailed analysis, and they were refereed as *qBWRB02-1-1* and *qBWRB02-1-2*, respectively (Table 4).

### Validation of genomic regions using newly developed KASP markers

To validate *qBWRB02-1-1*, 100 SNPs with the top ΔSNP-indexes were targeted for KASP marker development. The flanking 200 bp sequences of 27 SNPs were found to be specific when BLASTn to the Fuhuasheng reference genome. Finally, 11 KASP markers were successfully developed for *qBWRB02-1-1* (Table S8). Similarly, 100 SNPs with the top ΔSNP-indexes for *qBWRB02-1-2* were targeted for KASP marker development, and 15 KASP markers were successfully developed for *qBWRB02-1-2* (Table S8).

Genotyping of 268 RILs was performed with the 26 KASP markers and 4 SSR markers within the genomic region of *qBWRB02-1-1* and *qBWRB02-1-2*. Based on the results of the 30 markers, a genetic map was constructed with map length of 19.60 cM (Table S9). Single-marker analysis (SMA) confirmed the significant associations between BWR and the 30 markers across five environments (Table S9). Among the 13 markers for *qBWRB02-1-1*, the KASP marker CM014326.1_4160298 was the most significant, with LOD scores of 36.02–54.64. Among the 17 markers for *qBWRB02-1-2*, the KASP marker CM014326.1_6709822 was the most significant, with LOD scores of 15.19–22.97. CIM analysis indicated that the major and stable effects (49.43–59.90% PVE) of *qBWRB02-1-1* while the minor effects (3.96–6.35% PVE) of *qBWRB02-1-2* across five environments (Table 5).

**Table 5:** Validation of the genomic regions identified by QTL-seq approach on chromosome B02

QTL	Env^a^	Marker interval	Most significant marker	LOD value	Additive effect	PVE^b^ (%)
*qBWRB02-1-1*	HA2015	CM014326.1-4120413–AHGS2344	CM014326.1-4160298	36.65	24.98	59.37
	HA2016	CM014326.1-4120413–AHGS2344	CM014326.1-4160298	36.97	21.24	53.06
	HA2017	CM014326.1-4120413–AHGS2344	CM014326.1-4160298	38.22	22.94	52.40
	NC2015	CM014326.1-4120413–AHGS2344	CM014326.1-4160298	31.60	26.30	49.43
	NC2017	CM014326.1-4120413–AHGS2344	CM014326.1-4160298	47.09	23.71	59.99
	Mean	CM014326.1-4120413–AHGS2344	CM014326.1-4160298	56.85	24.02	68.86
*qBWRB02-1-2*	HA2015	CM014326.1-6556572–pPGseq11H1-1	CM014326.1-6709822	3.88	7.65	4.03
	HA2016	CM014326.1-6556572–pPGseq11H1-1	CM014326.1-6709822	4.22	7.19	4.43
	HA2017	CM014326.1-6556572–pPGseq11H1-1	CM014326.1-6709822	6.55	10.03	6.35
	NC2015	CM014326.1-6556572–pPGseq11H1-1	CM014326.1–6709822	4.62	9.74	5.19
	NC2017	CM014326.1-6556572–pPGseq11H1-1	CM014326.1-6709822	4.42	7.31	3.96
	Mean	CM014326.1-6556572–pPGseq11H1-1	CM014326.1-6709822	8.27	8.54	6.48

^a^Environment

^b^Phenotypic variation explained

**Fig. 4:**
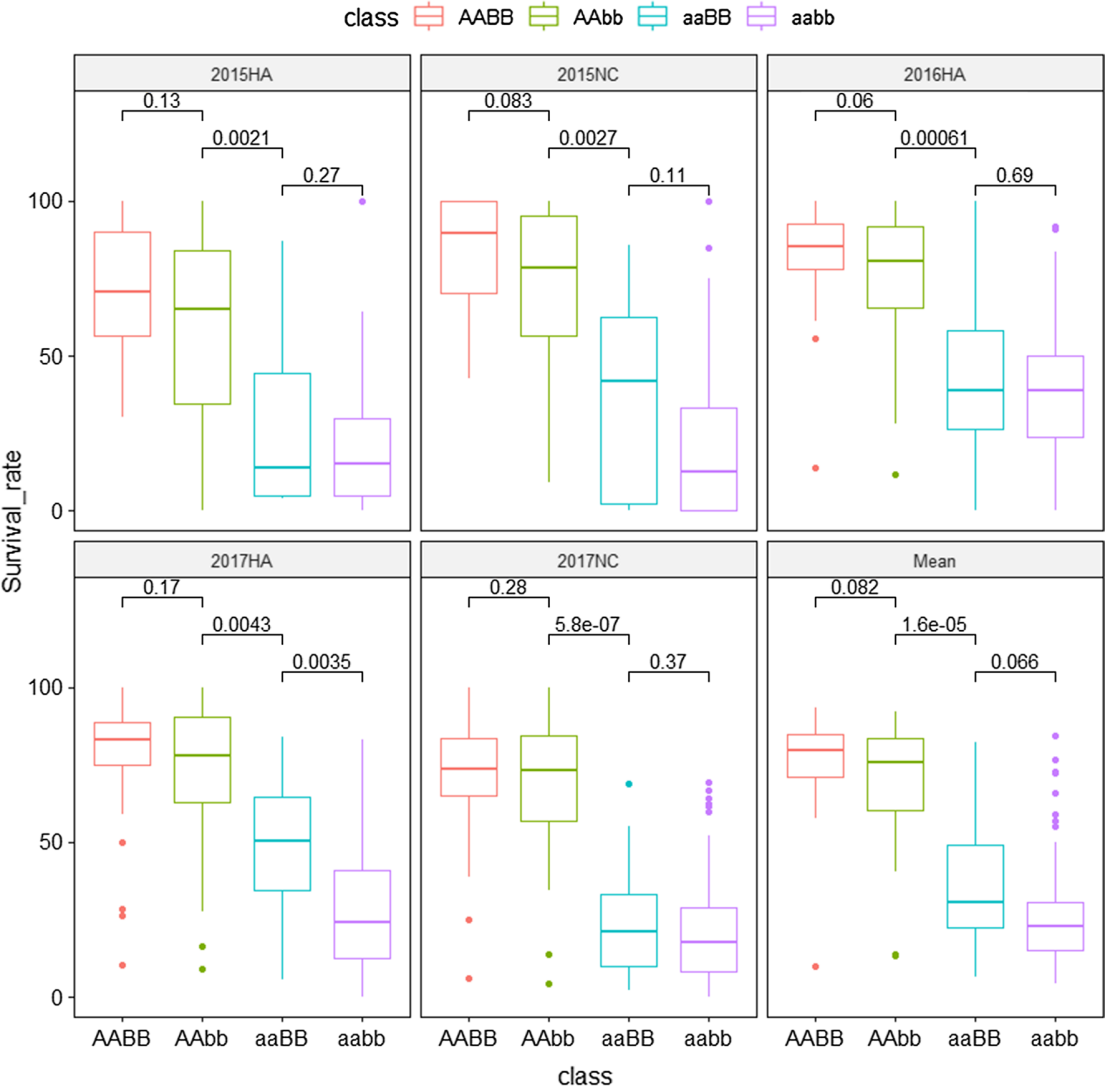
Boxplots for comparing the difference of survival rates between RILs with different genotypes. Boxplots were generated with the ggpubr package in R software. The *x*-axis represented the class of genotypes, while the *y*-axis represented values of survival rates (%). In each box, centerline shows the median; box limits indicate the 25th and 75th percentiles; whiskers extend 1.5 times the interquartile range from the 25th and 75th percentiles. The significant levels of differences between each group were illustrated as *P* values

To evaluate the combined effects of *qBWRB02-1-1* and *qBWRB02-1-2*, RILs were classified into four groups according to the genotypes of the KASP markers CM014326.1_4160298 and CM014326.1_6709822. The alleles of CM014326.1_4160298 and CM014326.1_6709822 from Zhonghua 6 were designated as “AA” and “BB,” respectively, while those from Xuhua 13 were designated as “aa” and “bb.” RILs with the AABB and AAbb genotypes showed significantly higher survival rates than those with the aaBB and aabb genotypes in all five environments (Fig. 4, Table S10). The average survival rate of RILs with the AABB genotypes was higher than RILs with the AAbb genotypes but not significantly. Similarly, the average survival rate of RILs with the aaBB genotypes was higher than RILs with the aabb genotype but not significantly. These results indicated that the resistance against bacterial wilt of Zhonghua 6 was mainly controlled by the *qBWRB02-1-1*.

### Putative candidate genes for BWR

The 1.43 Mb genomic region of *qBWRB02-1-1* had 205 effective SNPs. Of the 205 SNPs, 124 SNPs were identified irrespective of which parent was used as reference; however, 47 SNPs were specifically identified when the SP assembly was used as the reference while 34 SNPs were specifically identified when the RP assembly was used as the reference (Tables S11–12). Function annotation analysis of the 205 SNPs found that 30 SNPs were intergenic while the other 175 SNPs were located in genic regions of 50 putative genes. Among the 175 SNPs, 61 were located in 5 kb upstream, 3 in 5′ UTR, 23 intronic, 13 synonymous, 49 non-synonymous (1 resulted in stop codon) and 26 in 5 kb downstream. The 49 non-synonymous SNPs affected 16 putative candidate genes for BWR (Table 6). Notably, ten of the 16 candidate genes were predicted to code for disease resistance proteins, including *Ahy_B02g057386*, *Ahy_B02g057483*, *Ahy_B02g057484*, *Ahy_B02g057488*, *Ahy_B02g057489*, *Ahy_B02g057492*, *Ahy_B02g057531*, *Ahy_B02g057533*, *Ahy_B02g057534* and *Ahy_B02g057535* (Figure S11). The candidate gene *Ahy_B02g057524* codes for protein ENHANCED DISEASE RESISTANCE. The candidate genes *Ahy_B02g057494* and *Ahy_B02g057523* code for pentatricopeptide repeat superfamily protein. The candidate genes *Ahy_B02g057498* and *Ahy_B02g057528* code for serine threonine-protein phosphatase 7 long form homolog and hydroquinone glucosyltransferase, respectively. However, the candidate gene *Ahy_B02g057521* codes for protein with unknown function.

**Table 6:** Non-synonymous SNPs in putative candidate genes in the genomic region for bacterial wilt resistance on chromosome B02

Gene	Position (bp)	SP base	RP base	SB base	RB base	Amino acid change	Function	Reference assembly^a^
Ahy_B02g057386	2816632	T	G	T	G	Leu1443Phe	Disease resistance protein	Both
Ahy_B02g057483	3629422	C	T	C	T	Lys251Glu	Disease resistance protein	Both
	3629427	A	C	A	C	Arg249Ile		RP
	3629435	A	C	A	C	Arg246Ser		RP
	3629449	C	G	C	G	Val242Leu		RP
	3629461	A	G	A	G	Tyr238His		Both
	3629494	G	C	G	C	Leu227Val		RP
	3629516	A	T	A	T	Asp219Val		Both
	3629517	T	C	T	C	Asp219Val		Both
	3629533	C	G	C	G	Glu214Gln		Both
	3629545	C	G	C	G	Ala210Pro		Both
	3636318	T	C	T	C	Cys658Tyr		Both
	3637177	G	A	G	A	Trp375Arg		Both
Ahy_B02g057484	3652428	A	T	A	T	Lys556Met	Disease resistance protein	SP
	3652429	C	T	C	T	Lys556Glu		SP
	3654929	C	G	C	G	Asp119Glu		SP
Ahy_B02g057488	3676848	T	A	T	A	Thr1020Ser	Disease resistance protein	SP
	3677029	C	G	C	G	Asp959Glu		Both
Ahy_B02g057489	3695773	A	T	A	T	Ser519Thr	Disease resistance protein	Both
Ahy_B02g057492	3722570	C	G	C	G	Cys952Ser	Disease resistance protein	RP
	3723795	G	T	G	T	Leu544Ile		Both
	3723804	A	G	A	G	Ser541Pro		Both
	3724689	T	G	T	G	Lys246Gln		SP
Ahy_B02g057494	3729082	A	C	A	C	Asp275Glu	Pentatricopeptide repeat-containing protein	SP
	3729083	A	T	A	T	Leu276Met		SP
Ahy_B02g057498	3755618	C	T	C	T	Trp254^a^	Serine threonine-protein phosphatase 7 long form homolog	SP
Ahy_B02g057521	4025013	T	C	T	C	Gly65Glu	Unknown	RP
Ahy_B02g057523	4078542	C	T	C	T	Arg253Gln	Pentatricopeptide repeat-containing protein	SP
Ahy_B02g057524	4097658	T	A	T	A	Asp694Val	Protein ENHANCED DISEASE RESISTANCE	Both
Ahy_B02g057528	4124792	T	A	T	A	Glu35Val	hydroquinone glucosyltransferase	Both
Ahy_B02g057531	4150673	T	G	T	G	Phe79Leu	Disease resistance protein	Both
Ahy_B02g057533	4194809	T	G	T	G	Phe72Leu	Disease resistance protein	RP
Ahy_B02g057534	4196305	C	G	C	G	Thr13Arg	Disease resistance protein	Both
	4196326	T	A	T	A	Phe20Tyr		Both
	4196332	T	A	T	A	Phe22Tyr		SP
	4196338	A	G	A	G	Glu24Gly		SP
	4196344	T	G	T	G	Cys26Phe		SP
	4196346	C	G	C	G	Pro27Ala		Both
	4196403	G	C	G	C	Arg46Gly		Both
	4196539	T	G	T	G	Phe91Cys		Both
	4196850	T	G	T	G	Tyr195Asp		Both
Ahy_B02g057535	4236081	G	C	G	C	Asn36Lys	Disease resistance protein	Both
	4236149	G	A	G	A	His59Arg		RP
	4237744	A	C	A	C	Thr591Pro		RP
	4238099	C	T	C	T	Thr709Ile		RP
	4238107	A	G	A	G	Asn712Ala		RP
	4238108	A	C	A	C	Asn712Ala		RP
	4238109	T	G	T	G	Asn712Ala		RP
	4238112	C	G	C	G	Asn713Lys		RP
Ahy_B02g057721	6817695	T	C	T	C	Ser253Phe	Unknown	Both
Ahy_B02g057750	7160780	C	T	C	T	Trp97^a^	Serine threonine-protein phosphatase 7 long form homolog	Both
Ahy_B02g057762	7362813	G	T	G	T	Asp251Ala	transposon protein	Both
Ahy_B02g057766	7444948	C	T	C	T	Asn325Asp	glucose-6-phosphate 1-dehydrogenase	Both
Ahy_B02g057785	7664044	C	G	C	G	Arg191Thr	Mitochondrial glycoprotein family protein	RP
	7664059	G	A	G	A	Tyr196Cys		RP
Ahy_B02g057787	7681335	G	A	G	A	Arg338Gly	Mitochondrial glycoprotein family protein	RP
Ahy_B02g057789	7717654	T	C	T	C	Gly308Asp	Serine threonine-protein phosphatase 7 long form homolog	Both
Ahy_B02g057800	7912404	G	T	G	T	Val241Gly	replication protein A 70 kDa DNA-binding subunit E	Both
Ahy_B02g057801	7919763	T	G	T	G	Asn28Lys	pre-mRNA-processing factor 39-like isoform X1	Both
Ahy_B02g057808	8042482	C	T	C	T	Ser457Gly	Serine threonine-protein phosphatase 7 long form homolog	Both
Ahy_B02g057809	8059557	C	T	C	T	Cys248Arg	Serine threonine-protein phosphatase 7 long form homolog	Both
Ahy_B02g057838	8253903	C	T	C	T	Lys217Glu	ARID BRIGHT DNA-binding domain-containing protein	Both
	8254004	C	T	C	T	Asn183Ser		Both
Ahy_B02g057842	8318756	G	C	G	C	Ala692Gly	E3 ubiquitin-protein ligase RNF144A-like	SP
Ahy_B02g057850	8407952	C	T	C	T	Val123Ala	E3 ubiquitin-protein ligase RNF144A-like	Both
Ahy_B02g057860	8554265	G	T	G	T	Tyr329Asp	Unknown	Both
Ahy_B02g057386	2816632	T	G	T	G	Leu1443Phe	Disease resistance protein	Both

^a^Both indicated that the SNP was identified irrespective of parent used as reference. SP indicated that the SNP was identified specifically with the susceptible parent Xuhua 13 as reference. RP indicated that the SNP was identified specifically with the resistant parent Zhonghua 6 as reference

The 2.21 Mb genomic region of *qBWRB02-1-2* had 464 effective SNPs. Of the 464 SNPs, 433 SNPs were identified irrespective of parent used as reference; however, 12 SNPs were specifically identified when SP assembly was used as reference while 19 SNPs were specifically identified when RP assembly was used as reference (Table S11-12). Function annotation analysis of the 464 SNPs found that 160 SNPs were intergenic while the other 304 SNPs located in genic region of 112 putative genes. Among the 304 SNPs, 136 were located in 5 kb upstream, 6 in 5′ UTR, 33 intronic, 7 synonymous, 17 non-synonymous (1 resulted in stop codon), 3 in 3′ UTR and 102 in 5 kb downstream. Notably, the 17 non-synonymous SNPs affected 15 putative candidate genes for BWR (Table 6). Two of the 15 candidate genes, *Ahy_B02g057721* and *Ahy_B02g057860*, might code for protein with unknown function. The candidate genes *Ahy_B02g057842* and *Ahy_B02g057850* code for E3 ubiquitin-protein ligase RNF144A-like proteins. The candidate genes *Ahy_B02g057785* and *Ahy_B02g057787* code for mitochondrial glycoprotein family protein. The candidate genes *Ahy_B02g057766, Ahy_B02g057838, Ahy_B02g057762*, *Ahy_B02g057800* and *Ahy_B02g057801* code for glucose-6-phosphate 1-dehydrogenase, ARID BRIGHT DNA-binding domain-containing protein, transposon protein, replication protein A 70 kDa DNA-binding subunit E and pre-mRNA-processing factor 39-like isoform X1, respectively (Table 6).

## Discussion

Three high-quality reference genomes for both the subspecies of cultivated tetraploids were published in 2019 (Bertioli et al. 2019; Chen et al. 2019; Zhuang et al. 2019). Before this year, we had previously conducted QTL-seq analysis using diploid reference genomes (Bertioli et al. 2016); however, the availability of the tetraploid genome facilitated performing such analysis making it more precise and accurate. Prior to this study, although the genetic and genomic QTL analyses of BWR were performed for two resistant sources namely Yueyou 92 (Zhao et al. 2016) and Yuanza 9102 (Luo et al. 2019), the genetic basis of the majority resistant germplasm could not be dissected. Through the present study, we have successfully performed combination of genetic mapping and QTL-seq analysis using tetraploid reference genome and identified genomic regions, candidate genes and efficient markers for BWR in a promising resistant source Zhonghua 6.

### Construction of high-density genetic map in cultivated peanut

Constructing high-density genetic maps and performing genomic-wide QTL discovery has been the routine approach using bi-parental genetic populations in crop plants, including peanut. Among different marker systems, SSRs were widely used to construct genetic linkage maps and to map QTLs for disease resistance, drought tolerance, quality- and yield-related traits in cultivated peanut since 2009 (Varshney et al. 2009; Vishwakarma et al. 2017). In our previous study (Luo et al. 2018), a SSR-based genetic map of the RIL population derived from Xuhua 13 × Zhonghua 6 was constructed with 817 loci, which has been improved to 1002 loci in the present study. A total of 185 loci were selectively added to 16 of the 20 chromosomes according to the positions of SSRs in the reference genomes of *Arachis* (Bertioli et al. 2016, 2019). Compared to the reported SSR-based genetic maps (Huang et al. 2016; Yu et al. 2019), the loci number (1200) and density (1.83 cM/locus) of the present genetic map reached a fairly high level for performing precise QTL discovery. However, in recent times, the SNP markers have emerged as choice of markers due to their amenability for high throughput data generation and being cost-effective and time-saving (Pandey et al. 2016). The SNP-based genetic maps were constructed through multiple sequencing-based genotyping methods such as double-digest restriction-site-associated DNA sequencing (ddRADseq) (Zhou et al. 2014), genotyping-by-sequencing (GBS) (Dodia et al. 2019), specific length amplified fragment sequencing (SLAF-seq) (Hu et al. 2018; Li et al. 2019; Wang et al. 2018b) and whole-genome resequencing (Agarwal et al. 2018). These genetic maps were medium to high density as compared to sparsely dense SSR-based genetic maps. For example, a SNP-based linkage map for the same RIL population (Xuhua 13 × Zhonghua 6) was constructed with 2,595 SNP loci through ddRADseq (Liu et al. 2019). These SNP markers were classified into 20 linkage groups according to their RAD tags hits in the reference genomes of *A. duranensis* and *A. ipaensis* (Bertioli et al. 2016). Therefore, the published reference genomes of *Arachis* facilitated the construction of genetic maps in cultivated peanut, and the high-density SSR- and SNP-based genetic maps laid a foundation for the characterization of the genetic components controlling BWR.

### Genetic map-based QTL mapping and sequencing-based QTL-seq uncovers candidate genomic regions controlling BWR in a new resistant variety Zhonghua 6

Genetic factors play major roles in the determination of resistance to bacterial wilt in peanut, which has shown high (81.72%) broad-sense heritability in the RIL population of Yuanza 9102 (resistant) × Xuzhou 68–4 (susceptible) (Wang et al. 2018a). The present study also observed high broad-sense heritability for BWR, i.e., 71.07% based on plot mean and 93.72% based on entry mean, in another RIL population of Xuhua 13 (susceptible) × Zhonghua 6 (resistant). In previous studies, the BWR in peanut variety Yueyou 92 was found to be controlled by one major and stable QTL *qBW-1* plus one unstable QTL *qBW-2* (Zhao et al. 2016), and the BWR in Yuanza 9102 was also controlled by one major and stable QTL *qBWB02.1* plus three unstable minor QTLs (Wang et al. 2018a). In the present study, genome-wide QTL analysis identified one major and two minor QTLs (Table 1, Figure S3) using the improved SSR-based genetic map. The major QTL *qBWRB02-1* was stable in expression across five environments (53.93–78.86% PVE), while the two minor QTLs were only expressed in single environments (3.78–4.31% PVE). The major QTL *qBWRB02-1* was also identified using the SNP-based genetic map, while two additional minor QTLs (3.75–5.88% PVE) were identified in single environments (Table 2; Fig. 2; Figure S4). Therefore, major and stable QTLs for BWR exist in peanut, which will be valuable in the breeding of elite varieties with enhance resistance and agronomic traits.

Using the reference genome of the diploid ancestors of cultivated peanut (Bertioli et al. 2016), the QTL-seq approach has proved to be very successful in identifying candidate regions for major and stable QTL *qBWB02.1* in Yuanza 9102 (Luo et al. 2019). Recently, the genome sequences of the three cultivated peanut genotypes, including Fuhuasheng (Chen et al. 2019), Shitouqi (Zhuang et al. 2019) and Tifrunner (Bertioli et al. 2019), were published and made available to the peanut research community. Based on the pedigree of parental genotypes (Zhonghua 6 and Xuhua 13), the closest tetraploid genome sequence of Fuhuasheng was used as reference in QTL-seq pipeline (Figure S1) to identify the physical interval for the major QTL *qBWRB02-1*. In the previous report (Luo et al. 2019), there was significant difference of SNPs (164,522 vs. 243,380) identified using different parental assembly as reference. However, the number and SNPs identified using different parental assembly as reference were quite similar (303,290 vs. 320,522), indicating that the improvement of reference genome could make the QTL-seq more precise and accurate. Two adjacent genomic regions (2.81–4.24 Mb and 6.54–8.75 Mb) on chromosome B02 were identified within the confidential interval of *qBWRB02-1*, thus designated as *qBWRB02-1-1* and *qBWRB02-1-2*, respectively (Table 4; Fig. 3). Through validation with KASP markers, *qBWRB02-1-1* had major effects (49.43–68.86% PVE) while *qBWRB02-1-2* had minor effects (3.96–6.48% PVE); however, both of them were stable expressed across five environments (Table 5). Different from previous report (Luo et al. 2019), the QTL-seq approach could identify candidate regions not only for major QTL but also for minor QTL as long as it is stably expressed across environments.

### *qBWRB02-1-1* would be a novel and valuable QTL in breeding elite varieties with enhanced BWR

The major QTL *qBW-1* identified from Yueyou 92 might derive from Chinese landrace Xiekangqing (Zhao et al. 2016) and locate on chromosome B04 (Luo et al. 2019). Although the major QTL *qBWRB02.1* identified from Yuanza 9102 was located on chromosome B02, the resistant allele might derive from *A. diogoi* through interspecific hybridization according to pedigree analysis and it was absent in Zhonghua 6 and Yueyou 92 according to diagnostic marker detection (Luo et al. 2019). Based on the pedigree tracking, the source of *qBWRB02-1-1* identified on chromosome B02 from Zhonghua 6 in the present study was from a Chinese landrace Taishan Zhenzhu. The PVEs (49.43–68.86%) of *qBWRB02-1-1* were much higher than the previously reported *qBW-1* in Yueyou 92 (11.9–21.6% PVE) and *qBWRB02.1* in Yuanza 9102 (14.4–29.32% PVE). Therefore, we assumed that *qBWRB02-1-1* should be a novel and valuable QTL, and it is possible to further improve resistance level to bacterial wilt disease by pyramiding diverse resistant QTLs/genes from both wild and cultivated peanut.

### Candidate genes identified for *qBWRB02-1-1*

Through e-PCR of SSR markers and BLASTn of RAD tags, the physical interval of *qBWRB02-1-1* could be estimated but limited information was provided for the identification of candidate genes. Through QTL-seq, high-density SNPs were identified by resequencing of the parental genotypes and extreme pools. Function annotation analysis of non-synonymous SNPs variations identified 16 putative candidate genes for *qBWRB02-1-1* (Table 6). Ten candidate genes, including *Ahy_B02g057386*, *Ahy_B02g057483*, *Ahy_B02g057484*, *Ahy_B02g057488*, *Ahy_B02g057489*, *Ahy_B02g057492*, *Ahy_B02g057531*, *Ahy_B02g057533*, *Ahy_B02g057534* and *Ahy_B02g057535*, were predicted to code for disease resistance proteins which play keys role in the plant immune system (Jones and Dangl 2006; Spoel and Dong 2012). These proteins shared typical Rx N-terminal (Rx_N) domain (Hao et al. 2013), nucleotide binding site (NBS) domain (van Ooijen et al. 2008), leucine-rich repeat (LRR) domain (Sanseverino et al. 2010) of R-genes (Figure S11). Notably, maximum number of non-synonymous SNPs (12) was identified in the *Ahy_B02g057483* gene, flowed by *Ahy_B02g057534* (9) and *Ahy_B02g057535* (8). The gene *Ahy_B02g057524* codes homolog of EDR4 which plays a negative role in disease resistance (Wu et al. 2015). The candidate genes *Ahy_B02g057494* and *Ahy_B02g057523* code for pentatricopeptide repeat superfamily proteins, whose knockdown mutants displayed more severe disease symptoms when challenged by pathogenic bacteria (Park et al. 2014). The gene *Ahy_B02g057498* codes homolog of serine threonine-protein phosphatase 7 long form protein which might take part in hypersensitive response (Zhou et al. 1995). The identified non-synonymous SNPs in the *Ahy_B02g057498* gene resulted in early termination of protein translation. The gene *Ahy_B02g057528* codes homolog of hydroquinone glucosyltransferase whose suppressed expression might improve resistance to bacteria (Park et al. 2011). Therefore, the above genes should be targeted as candidates for fine mapping and functional validation.

In conclusion, the present study identified two novel and adjacent QTLs on chromosome B02 controlling resistance to bacterial wilt disease in peanut-resistant variety Zhonghua 6. Promising candidate genes were identified in these intervals but cannot be overemphasized until validated through fine mapping and/or complementation tests. These genomic regions and validated markers intervals would be of great value for marker-assisted selection to improve BWR in future peanut breeding.

## Electronic supplementary material

Below is the link to the electronic supplementary material.

Figure S1 QTL-seq approach used for mapping bacterial wilt resistance in peanut (PDF 262 kb)

Figure S2 Graphical presentation of the improved SSR-based genetic maps constructed in the RIL population derived from Xuhua 13 and Zhonghua 6 (PDF 53 kb)

Figure S3 Genome-wide overview of QTLs for bacterial wilt resistance identified using the improved SSR-based genetic map (TIF 627 kb)

Figure S4 Genome-wide overview of QTLs for bacterial wilt resistance identified using the SNP-based genetic map (TIF 658 kb)

Figure S5 Genome-wide SNP-index plots of resistant bulk with the susceptible parent Xuhua 13 as reference (PDF 964 kb)

Figure S6 Genome-wide SNP-index plots of susceptible bulk with the susceptible parent Xuhua 13 as reference (PDF 871 kb)

Figure S7 The ΔSNP-index plot obtained by subtraction of susceptible bulk SNP-index from resistant bulk SNP-index using the susceptible parent Xuhua 13 reference (PDF 931 kb)

Figure S8 Genome-wide SNP-index plots of susceptible bulk with the resistant parent Zhonghua 6 as reference (PDF 1019 kb)

Figure S9 Genome-wide SNP-index plots of resistant bulk with the resistant parent Zhonghua 6 as reference (PDF 935 kb)

Figure S10 The ΔSNP-index plot obtained by subtraction of susceptible bulk SNP-index from resistant bulk SNP-index using the resistant parent Zhonghua 6 as reference (PDF 953 kb)

Figure S11 The conserved domains encoded by the identified candidate genes in the genomic region of *qBWRB02-1-1*. (PDF 407 kb)

Table S1 Result of ANOVA for BWR of the RIL population across five environments. Table S2 Information of 180 SSR markers newly genotyped in the RIL population. Table S3 Genotyping data of the 185 newly genotyped SSR loci in the RIL population. Table S4 Genetic linkage map constructed based on 1002 polymorphic SSR loci in the RIL population. Table S5 Description of the improved genetic map constructed based on SSR markers. Table S6 Details on whole genome re-sequencing data for QTL-seq. Table S7 Chromosome-wise SNPs distribution between extreme bulks for bacterial wilt resistance. Table S8 Details of newly developed KASP markers. Table S9 Single-marker analysis for bacterial wilt resistance using validated KASP markers. Table S10 Phenotypic effect of the two adjacent QTLs on chromosome B02 for bacterial wilt resistance in the RIL population. Table S11 Effective SNPs identified for bacterial wilt resistance using the susceptible parent Xuhua 13 assembly as reference. Table S12 Effective SNPs identified for bacterial wilt resistance using the resistant parent Zhonghua 6 assembly as reference (XLSX 379 kb)
