# Supplementary figures and images for: Discovery of two novel and adjacent QTLs on chromosome B02 controlling resistance against bacterial wilt in peanut variety Zhonghua 6
Source: Theor Appl Genet. 2020 Jan 24;133(4):1133–48. doi: 10.1007/s00122-020-03537-9 (PMC7064456; doi:10.1007/s00122-020-03537-9)

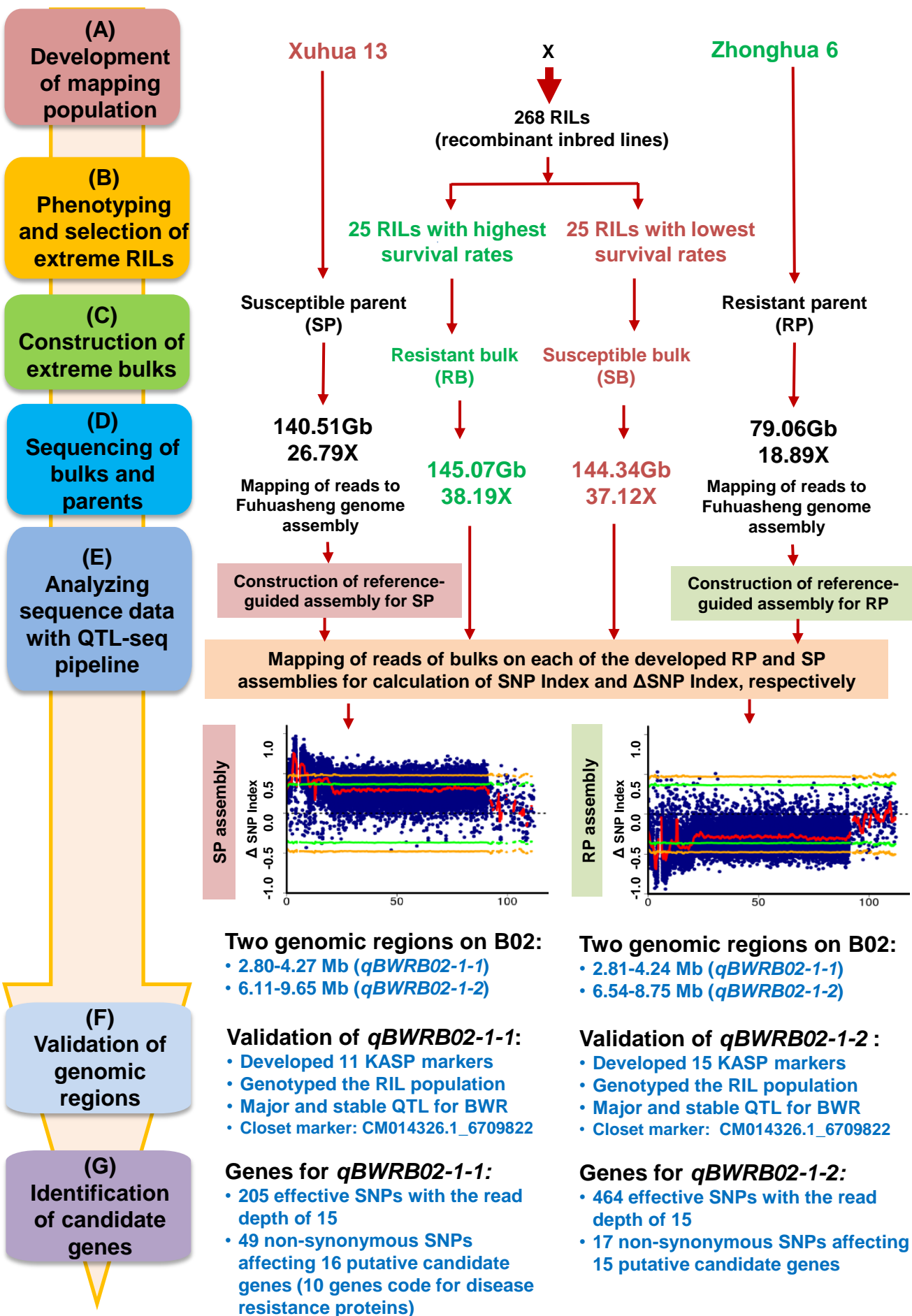

**Figure S QTL-seq approach used for mapping bacterial wilt resistance in peanut.**

Supplement: Supplementary file 1 — QTL-seq approach used for mapping bacterial wilt resistance in peanut (PDF 262 kb) [file 122_2020_3537_MOESM1_ESM.pdf]

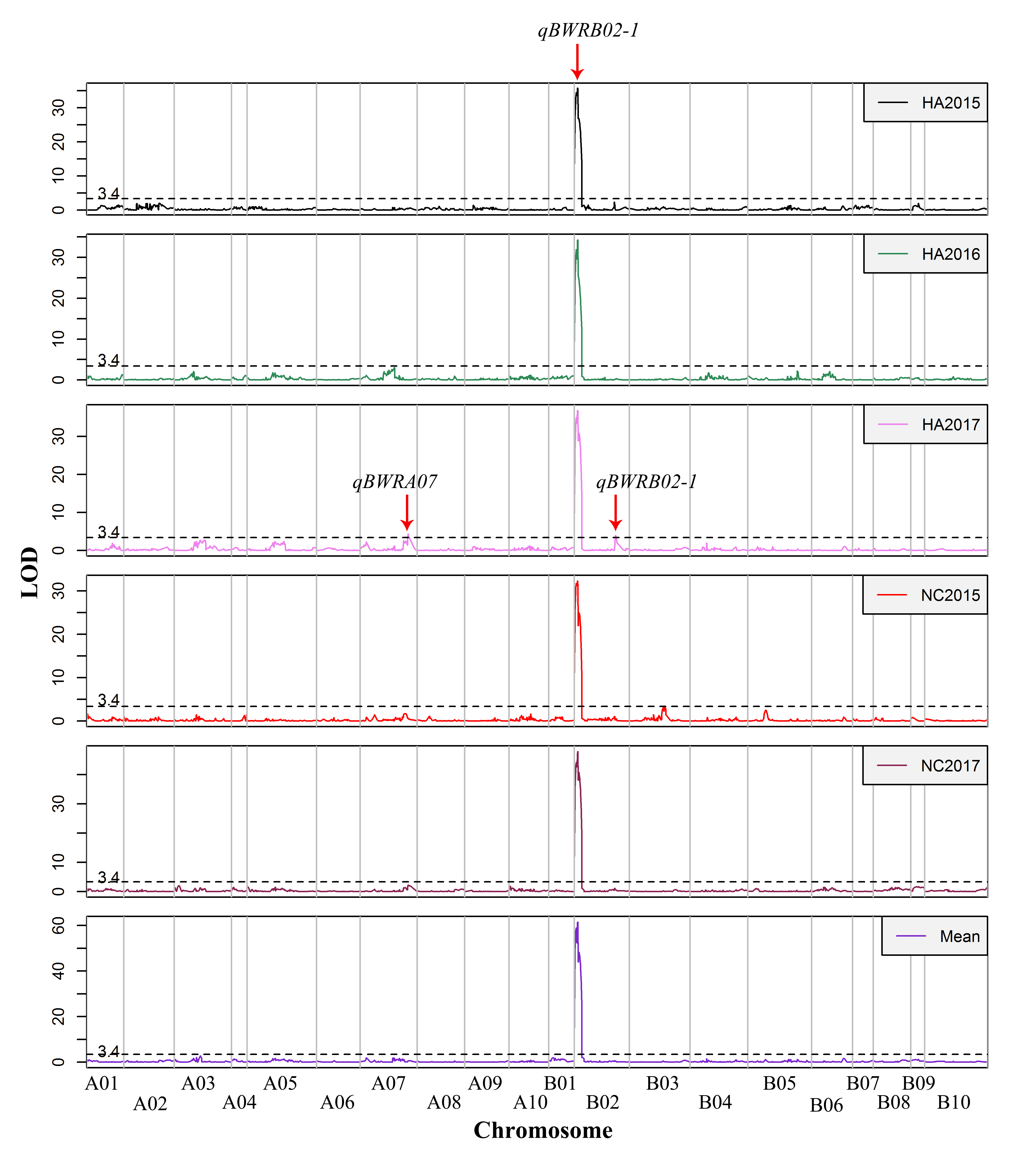

Supplement: Supplementary file 3 — Genome-wide overview of QTLs for bacterial wilt resistance identified using the improved SSR-based genetic map (TIF 627 kb) [file 122_2020_3537_MOESM3_ESM.tif]

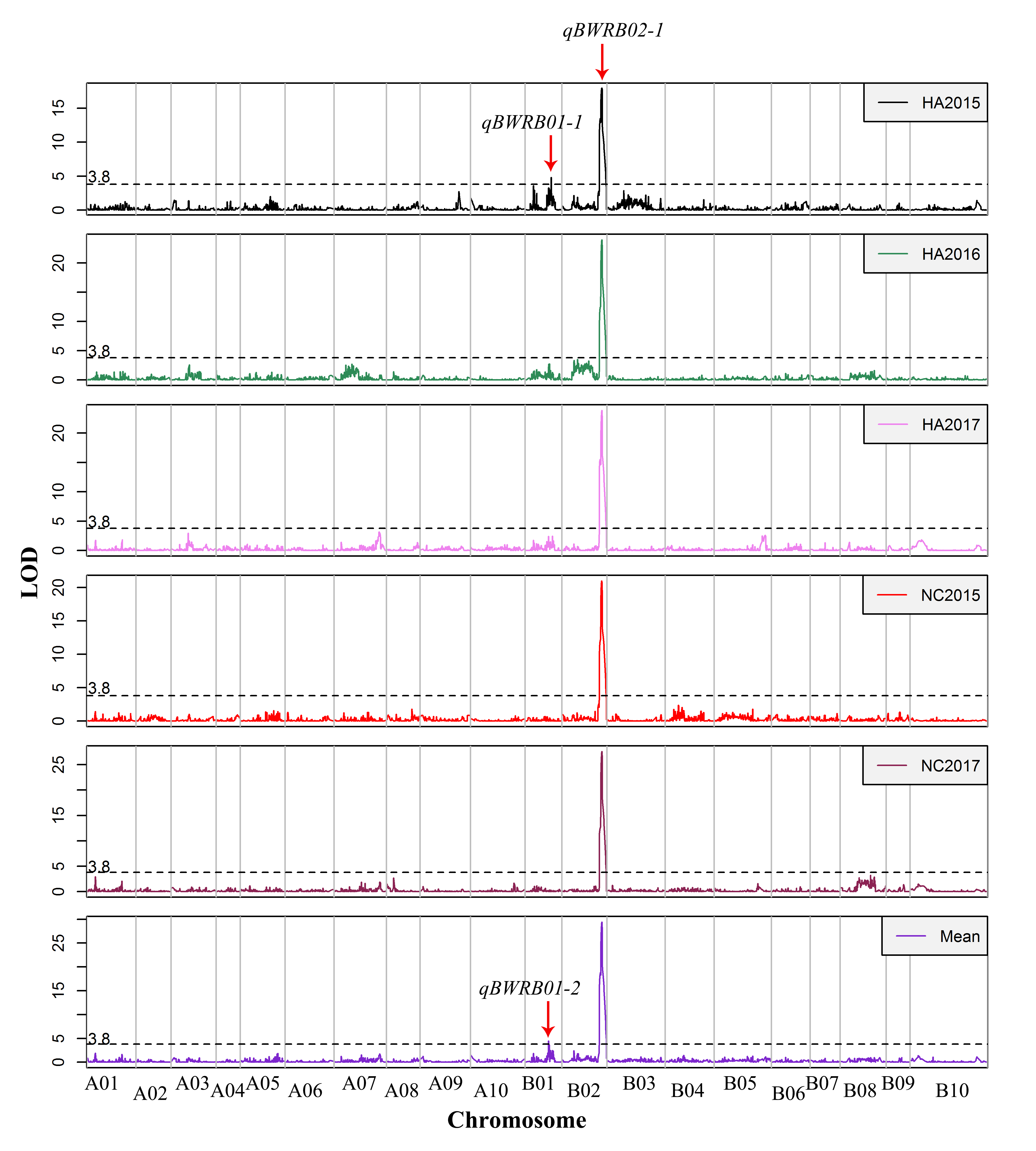

Supplement: Supplementary file 4 — Genome-wide overview of QTLs for bacterial wilt resistance identified using the SNP-based genetic map (TIF 658 kb) [file 122_2020_3537_MOESM4_ESM.tif]
