# Supplementary material for: Discovery of two novel and adjacent QTLs on chromosome B02 controlling resistance against bacterial wilt in peanut variety Zhonghua 6
Source: Theor Appl Genet. 2020 Jan 24;133(4):1133–48. doi: 10.1007/s00122-020-03537-9 (PMC7064456; doi:10.1007/s00122-020-03537-9)
Supplement: Supplementary file 2 — Graphical presentation of the improved SSR-based genetic maps constructed in the RIL population derived from Xuhua 13 and Zhonghua 6 (PDF 53 kb) [file 122_2020_3537_MOESM2_ESM.pdf]

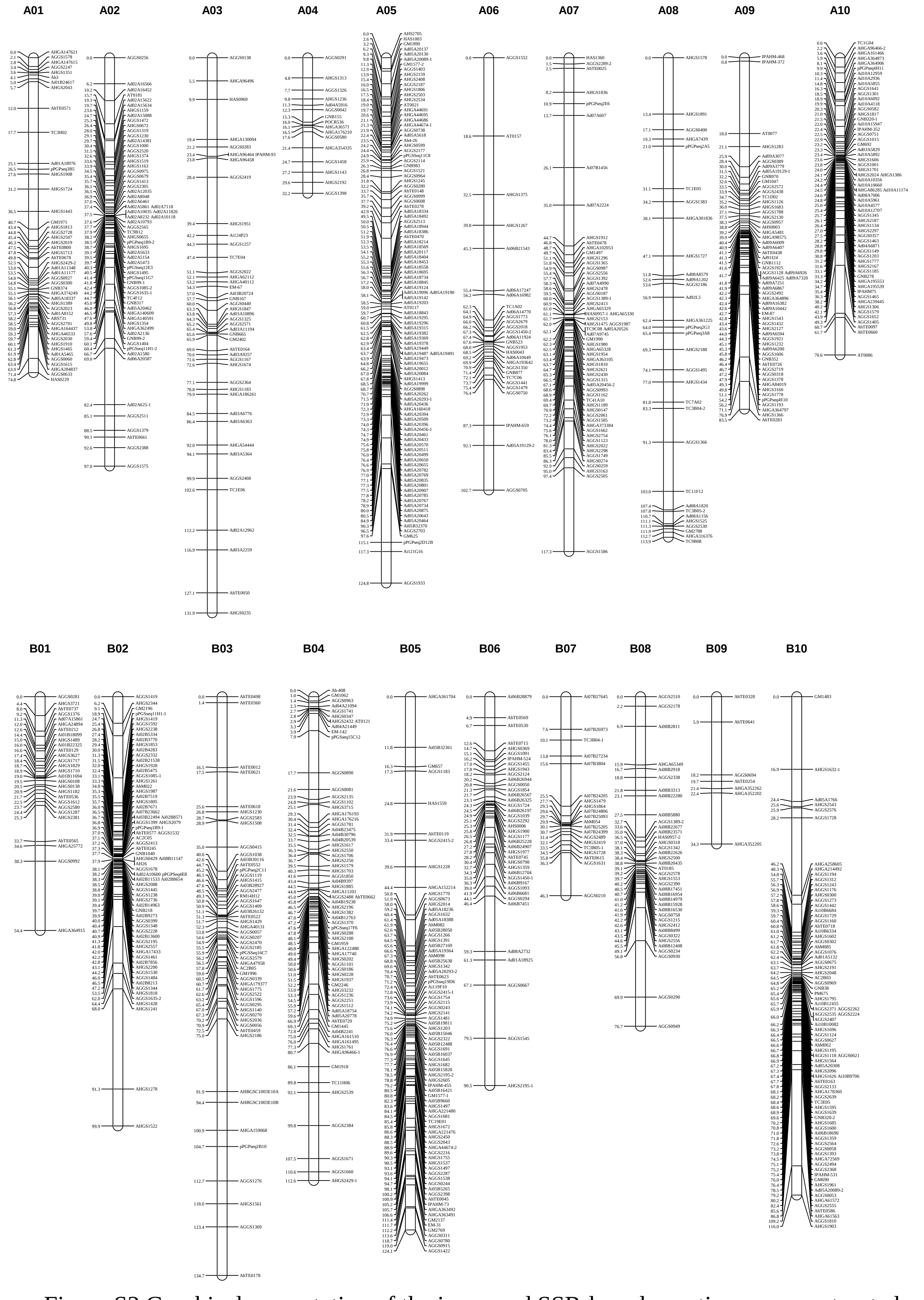

Figure S2 Graphical presentation of the improved SSR-based genetic maps constructed in the RIL population derived from Xuhua 13 and Zhonghua 6
