# Supplementary material for: Discovery of two novel and adjacent QTLs on chromosome B02 controlling resistance against bacterial wilt in peanut variety Zhonghua 6
Source: Theor Appl Genet. 2020 Jan 24;133(4):1133–48. doi: 10.1007/s00122-020-03537-9 (PMC7064456; doi:10.1007/s00122-020-03537-9)
Supplement: Supplementary file 5 — Genome-wide SNP-index plots of resistant bulk with the susceptible parent Xuhua 13 as reference (PDF 964 kb) [file 122_2020_3537_MOESM5_ESM.pdf]

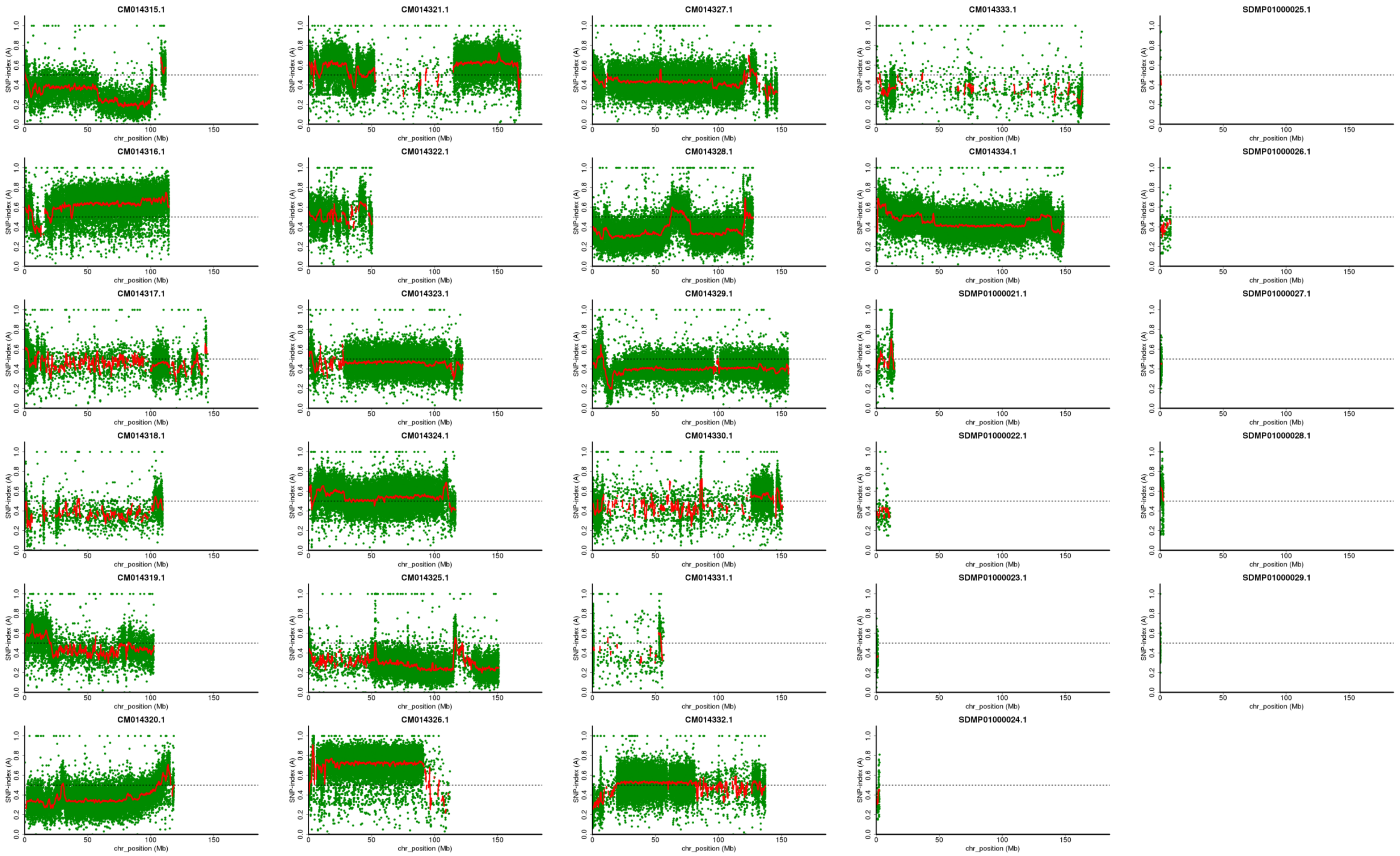

Figure S5 Genome-wide SNP-index plots of resistant bulk with the susceptible parent Xuhua 13 as reference. Red lines indicate the sliding window average of 1 Mb interval with 50 kb increment for SNP-index.
