# Supplementary material for: Discovery of two novel and adjacent QTLs on chromosome B02 controlling resistance against bacterial wilt in peanut variety Zhonghua 6
Source: Theor Appl Genet. 2020 Jan 24;133(4):1133–48. doi: 10.1007/s00122-020-03537-9 (PMC7064456; doi:10.1007/s00122-020-03537-9)
Supplement: Supplementary file 7 — The ΔSNP-index plot obtained by subtraction of susceptible bulk SNP-index from resistant bulk SNP-index using the susceptible parent Xuhua 13 reference (PDF 931 kb) [file 122_2020_3537_MOESM7_ESM.pdf]

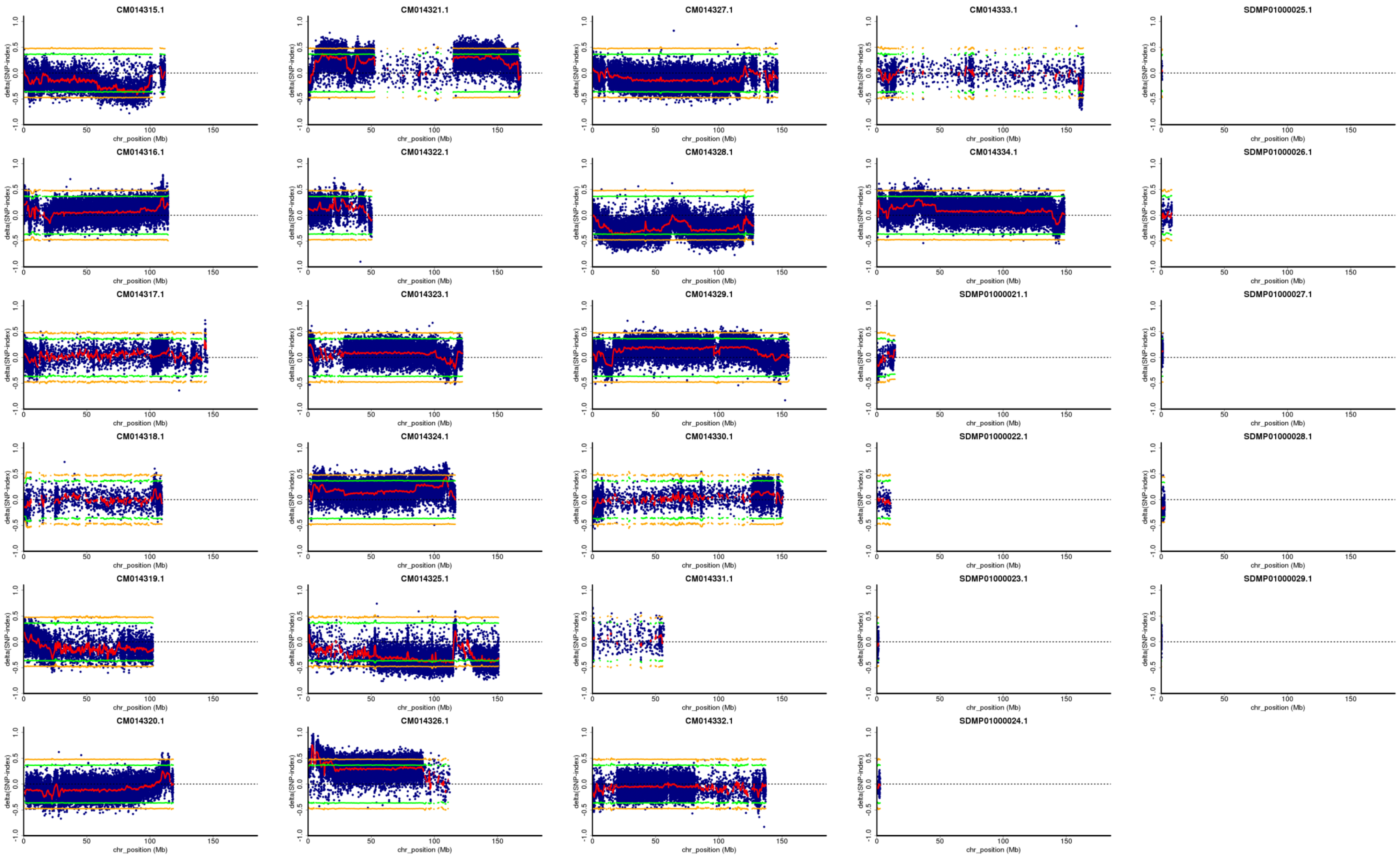

Figure S7 The ΔSNP-index plot obtained by subtraction of susceptible bulk SNP-index from resistant bulk SNP-index using the susceptible parent Xuhua 13 reference. Statistical confidence intervals under the null hypothesis of no QTL are shown (green: $P < 0.05$; orange: $P < 0.01$).
