# Supplementary material for: Discovery of two novel and adjacent QTLs on chromosome B02 controlling resistance against bacterial wilt in peanut variety Zhonghua 6
Source: Theor Appl Genet. 2020 Jan 24;133(4):1133–48. doi: 10.1007/s00122-020-03537-9 (PMC7064456; doi:10.1007/s00122-020-03537-9)
Supplement: Supplementary file 11 — The conserved domains encoded by the identified candidate genes in the genomic region of qBWRB02-1-1. (PDF 407 kb) [file 122_2020_3537_MOESM11_ESM.pdf]

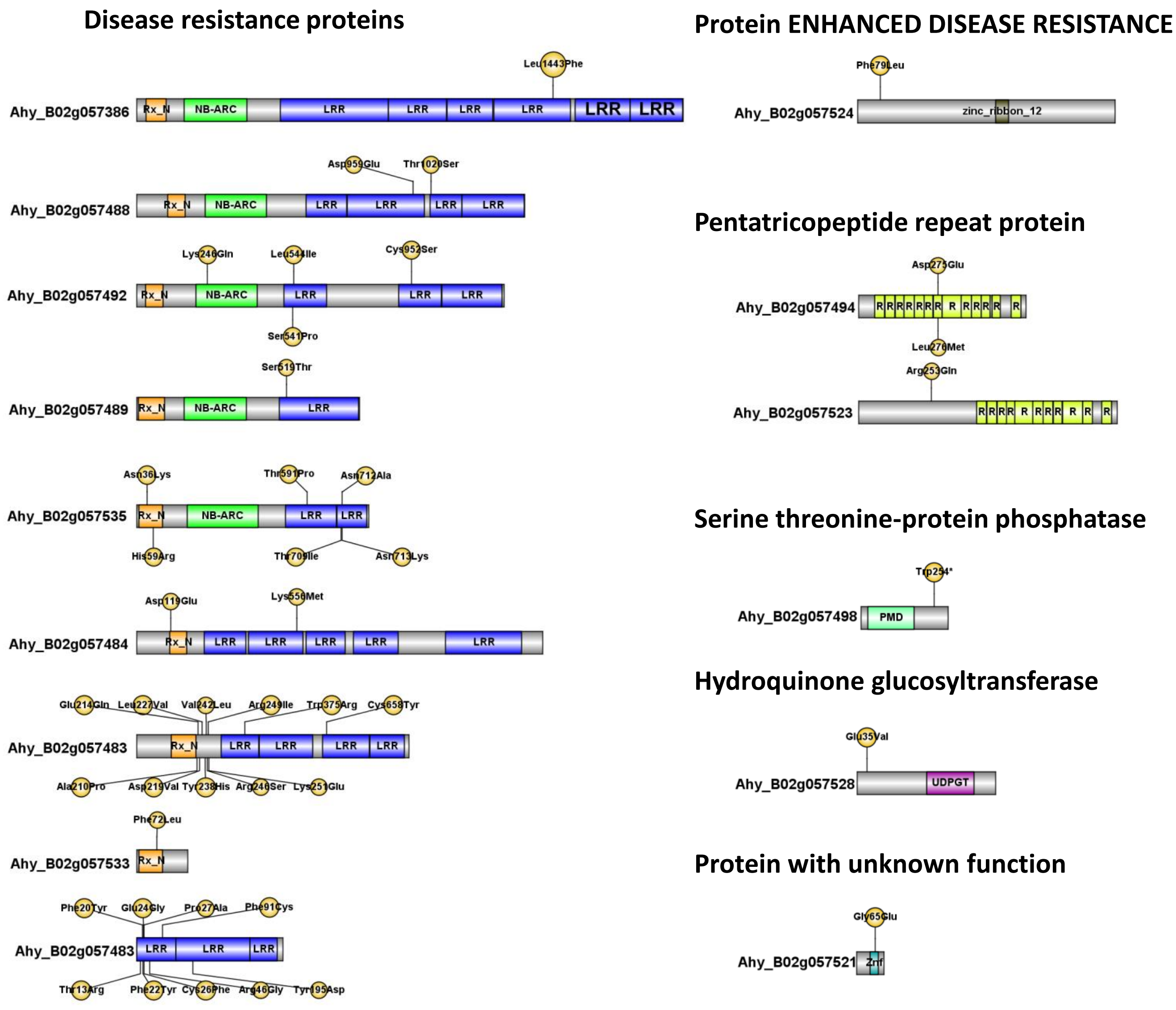

Figure S11 The conserved domains encoded by the identified candidate genes in the genomic region of *qBWRB02-1-1*. Rx_N: Rx N-terminal domain. NB-ARC: Nucleotide binding site domain. LRR: Leucine-rich repeat domain. R: Pentatricopeptide repeat domain. PMD: Plant mobile domain. Znf: Zinc finger domain. UDPGT: UDP-glucuronosyl/UDP-glucosyltransferase. Zinc_ribbon_12: Probable zinc-ribbon domain, plant. The positions of amino acid changes caused by non-synonymous SNPs were shown in yellow color.
